# Targeting the JAK/STAT Signaling Pathway in Breast Cancer: Leaps and Hurdles

**DOI:** 10.3390/biomedicines13123061

**Published:** 2025-12-12

**Authors:** Simon Aho, Clio Coste, Luiza Purcari, Olivier Trédan, Coralie Poulard, Benoite Mery, François-Michel Boisvert, Muriel Le Romancer

**Affiliations:** 1 Université de Lyon, F-69000 Lyon, France; simon.aho@lyon.unicancer.fr (S.A.); clio.coste@lyon.unicancer.fr (C.C.); luiza.purcari@lyon.unicancer.fr (L.P.); olivier.tredan@lyon.unicancer.fr (O.T.); coralie.poulard@lyon.unicancer.fr (C.P.); benoite.mery@lyon.unicancer.fr (B.M.); 2 Inserm U1052, Centre de Recherche en Cancérologie de Lyon, F-69000 Lyon, France; 3 CNRS UMR5286, Centre de Recherche en Cancérologie de Lyon, F-69000 Lyon, France; 4 Oncology Department, Centre Leon Bérard, F-69008 Lyon, France; 5 Department of Immunology and Cell Biology, Faculty of Medicine and Health Sciences, Université de Sherbrooke, Sherbrooke, QC J1H 5H3, Canada; francois.michel.boisvert@usherbrooke.ca

**Keywords:** breast cancer, JAK, STAT, signaling, inhibitors

## Abstract

The JAK/STAT (Janus kinase/signal transducer and activator of transcription) signaling pathway transfers signals at the surface of cell membranes to the nucleus, triggering the expression of a myriad of factors implicated in immunity, cell proliferation, and apoptosis. Owing to this central role in cell homeostasis, its dysregulation is extensively reported in tumorigenesis, particularly in hematological cancers, justifying the development of specific inhibitors. It has more recently also been implicated in the development of solid cancers, including breast cancer. However, so far, clinical trials testing drugs targeting actors of JAK/STAT signaling yielded disappointing results, advocating in favor of a better understanding of this pathway in breast cancer. Herein, we exhaustively reviewed the current tools available to target this pathway in clinical trials and we offer several perspectives to gain further insight into the role of JAK2 in breast cancer and more particularly in the resistance to endocrine therapy in hormone-dependent breast cancers.

## 1. Introduction

**Figure 1:**
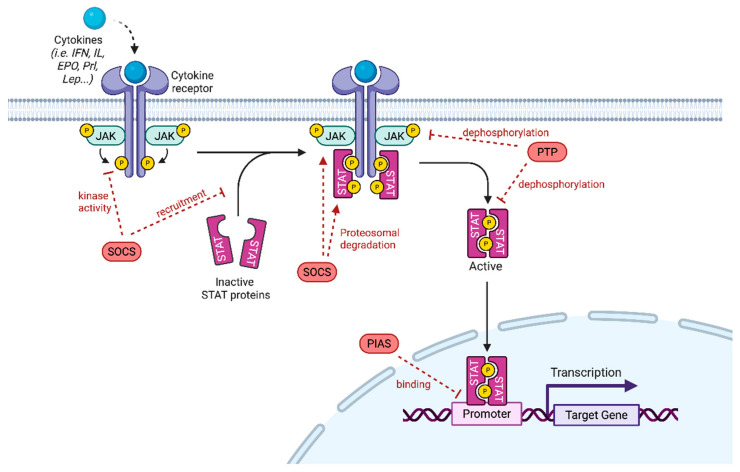
**JAK/STAT Signaling pathway.** Cytokines bind to their receptors, leading to receptor dimerization and recruitment of associated JAK. JAK activation includes JAK autophosphorylation and phosphorylation of the receptors that form docking sites for STAT. STAT are phosphorylated and they subsequently form homo- or heterodimers. STAT dimers enter into the nucleus, bind to DNA and regulate transcription. JAK/STAT signaling pathway can be negatively regulated by PIAS (protein inhibitor of activated STAT), PTP (protein tyrosine phosphatase) or SOCS (suppressor of cytokine signaling). PIAS inhibits STAT binding to DNA through its interaction with STAT dimers. PTP negatively regulates JAK/STAT signaling by dephosphorylating the STAT dimer and JAK. SOCS inhibits JAK/STAT signaling by inhibiting JAK kinase activity after direct binding to JAK, blocking STAT recruitment after binding to a tyrosine kinase receptor and by promoting JAK or STAT proteasomal degradation (adapted from Hu et al. [2]).

The JAK/STAT pathway plays an essential role in cellular proliferation and differentiation, organ development and immune homeostasis. Its dysregulation has been associated with tumorigenesis, by promoting cell proliferation, inducing anti-apoptotic activity, stem cell maintenance, immune suppression and angiogenesis. The JAK/STAT pathway is a complex network activated by over 50 cytokines and growth factors, such as interferons and interleukins [1]. Upon receptor activation, JAK proteins are recruited triggering their phosphorylation, which in turn induces the binding of STAT proteins and their phosphorylation on tyrosine residues. STAT proteins then dimerize, modify their conformation and enter the nucleus where they bind to DNA elements to regulate the transcription of specific target genes (Figure 1).

Aberrant JAK/STAT activation was described in the development of various cancer subtypes, from hematological malignancies to solid tumors such as hepatocarcinoma, prostate and breast cancer (BC). Furthermore, pathway dysfunctions are thought to be responsible, to some extent, for drug resistance and immune evasion of cancer cells. Indeed, it was reported that, among the STAT proteins, STAT3 plays a crucial role in cell cycle and apoptosis, therefore promoting by its constitutive activation oncogenesis, metastasis and even development of drug resistance.

Many JAK inhibitors displaying clinical efficacy have been and are being developed, though there are currently no approved drugs that selectively target STAT3 in the field of cancer treatment [2]. Numerous trials are ongoing in preclinical or clinical phases, with some promising results [3]. It is essential to note that JAK phosphorylates other substrates than STAT, notably in the nucleus, highlighting a more complex signaling pathway that must be taken into account to better target the oncogenic properties of JAK proteins with JAK inhibitors (JAKi), as these sometimes display limited efficacy in treating cancers.

Here, focusing on BC, we will provide current insights into the regulation and targeting of JAK/STAT signaling in solid cancers.

## 2. The JAK/STAT Signaling Pathway

JAK proteins are non-receptor tyrosine kinases activated when cytokines or growth factors bind to their receptors, which then phosphorylate the cytoplasmic part of the receptors creating docking sites for SH2-containing signaling proteins such as STAT (Figure 1).

JAK kinases include JAK1, JAK2, JAK3 and TYR2. While JAK3 is only expressed in endothelial, vascular smooth muscle cells and the lymphatic and bone marrow [4,5], the other members are ubiquitously expressed. JAK1 and JAK2 have been extensively studied and seem to play the most predominant role, as mice knocked out (KO) for JAK1 exhibit severe lymphocyte damage and neurological disease [6], and JAK2 KO mice die in utero due to impaired hematopoietic functions [7]. Conversely, JAK3 KO mice are defective in lymphocyte production but can survive in the absence of pathogens [8], while cytokine signaling is not completely lost in TyK2 KO mice [9].

JAK1 is phosphorylated by three families of receptors (cytokine receptors with the ƔC receptor subunit, class II cytokine receptors and receptors with a gp130 subunit) and can phosphorylate all STATs [10]. JAK2 similarly to JAK1 can be phosphorylated by class II cytokine-receptors and by the gp130 receptor family. In addition, it participates in the signal transduction of the growth hormone receptor and the prolactin receptor [11]. Approximately 20 phosphorylation residues have been identified. Tyr1007-1008 in the activation loop of the kinase domain is the major site of autophosphorylation and a prerequisite for its catalytic activation [7]. In the absence of a ligand, JAK2 is constitutively phosphorylated on Ser523 to inhibit its activation [12].

The STAT family comprises seven members: STAT1, STAT2, STAT3, STAT4, STAT5a, STAT5b and STAT6 involved in cellular signaling and gene regulation. STAT2, STAT4 and STAT6, are activated by interferons and interleukins and regulate the differentiation of T cells. Conversely, other STATs can also be activated by the growth hormone and the prolactin receptor, and are predominantly implicated in the regulation of tumor growth. For instance, STAT1 was shown to reduce cell growth by promoting the expression of p21 and p27 or inhibiting the expression of c-myc and cyclin [13]. STAT1 can also promote apoptosis by inducing Fas, BCL2 and BCLXL expression [14,15]. However, in certain contexts, STAT1 promotes tumor growth. STAT3 and STAT5 are often associated with oncogenic processes such as proliferation and survival. STAT5 KO mice present defects in the development of the mammary gland and milk secretion and in the production of GH [16].

Given that JAK/STAT signaling is dampened by several mechanisms, the lack of negative regulation may result in disorders including cancers. Such regulators include protein tyrosine phosphatases (PTPs) and proteins that physically suppress signal generation such as suppressors of cytokine signaling (SOCS) and protein inhibitors of activated STAT (PIAS) (Figure 1). It should be noted that JAK/STAT signaling is also regulated by cross talks with other pathways. As this event occurs mainly at the level of STAT proteins, this point will not be further developed herein (for a review see Hu et al. [2]). The SOCS family comprises 8 members SOCS1 1 to 7 and CIS. Activated STATs induce the expression of SOCS proteins that bind to phosphorylated JAK and its receptor to negatively regulate downstream signaling by impeding the binding of STATs but also by triggering their degradation. For instance, upon SOCS3 binding, the substrate-binding groove of JAK is hidden [17]. SOCS1 can inhibit the tyrosine activity of JAK through its KIR domain [18]. SOCSs also interact with cullin 5 to undergo polyubiquitination and trigger proteasomal degradation of JAKs and STATs [19].

The PIAS family contains four members acting as transcriptional regulators (PIAS1 to PIAS4). PIASs only interact with STAT dimers after being phosphorylated by JAK. They act by blocking the DNA-binding capacity of STAT [20], but also by promoting their sumoylation via their E3 ligase activity [21]. Thus, JAK/STAT activity increases when PIAS is knocked down leading to the formation of hematological tumors [22]. PTPs can also modulate JAK/STAT signaling by dephosphorylating JAK and STATs or their upstream receptors. The transmembrane PTP CD45 can inhibit IL3-mediated cell proliferation by inhibiting JAK2 phosphorylation [23]. SH-PTP1 can remove the phosphorylation on Y429 of the EPO receptor, mediating the inactivation of JAK2 [24]. SHP1 was also shown to dephosphorylate STAT5b. Nuclear TC45 inactivates STAT by dephosphorylation [25].

### Post-Translational Modifications in JAK/STAT Signaling

One of the key events regulating JAK/STAT signaling is post-translational modifications (PTMs). These modifications activate/inactivate proteins, including those involved in the JAK/STAT pathway, and their dysregulation is associated with various cancers. The most extensively studied PTM in the JAK/STAT pathway is phosphorylation, which is required for the activation of STAT proteins. Phosphoproteomics has provided detailed maps of phosphorylation sites on STAT3 and STAT5, identifying cancer-specific phosphorylation patterns that contribute to aberrant signaling [26]. For instance, in breast cancer (BC), hyperphosphorylation of STAT3 on the tyrosine residue Y705 has been linked to poor prognosis and increased metastatic potential [27]. It was also reported that peptides phosphorylated by members of the JAK-STAT and PKC signaling pathways are subjected to extensive kinase activity in chemotherapy-resistant MCF7 luminal cells. This observation led to the treatment of cells with tofacitinib, an FDA-approved JAK inhibitor, rendering chemoresistant cells sensitive to chemotherapy by inducing apoptosis when used in conjunction with Doxorubicin [28].

In addition to phosphorylation, other PTMs such as ubiquitination and acetylation have been identified in JAK/STAT signaling components. Proteomic studies have shown that STAT proteins can be ubiquitinated, thus regulating their stability and degradation. Dysregulation of ubiquitination has been linked to prolonged STAT activation in some cancers [29]. These findings highlight the importance of exploring a wider range of PTMs in JAK/STAT signaling, as they may reveal new therapeutic targets.

Proteomic studies have significantly advanced our understanding of the JAK/STAT pathway in cancer, providing insights into protein-level alterations and signaling mechanisms that drive oncogenesis. By employing advanced proteomic techniques such as mass spectrometry and phosphoproteomics, researchers identified key proteins, PTMs, and networks of interactions involved in JAK/STAT dysregulation. These discoveries have important implications for cancer diagnosis, prognosis, and treatment, particularly in identifying new therapeutic targets and understanding resistance to existing therapies. As proteomic technologies continue to evolve, future studies are expected to provide even more detailed insights into the complexities of the JAK/STAT pathway, ultimately improving cancer treatment strategies.

## 3. JAK/STAT in Hematological Malignancies

Activation of the JAK/STAT cascade is widely explored in the field of myeloproliferative neoplasms (MPNs) owing to acquired somatic mutations. JAK2 is the most frequently mutated protein in these MPNs, and the degree of substitution of valine to phenylalanine in JAK2^V617F^ is associated with the severity of the disease. Within the MPN subtypes, JAK2^V617F^ is present in up to 90% of polycythemia vera and about 50% of essential thrombocythemia and primary myelofibrosis. Other mutated proteins that have an essential role in promoting MPN development are calreticulin, a chaperone protein involved in protein folding, and the thrombopoietin receptor, MPL. JAK-STAT mutations in leukemia were initially believed to originate from MPNs. In 1995, the role of STAT1, STAT3 and STAT5 in de novo leukemia development was established. Among the STAT proteins, STAT5 majorly impacts the evolution of lymphomyeloid precursors. STAT1 was reported to act as an oncogenic promoter. The dysfunction of STAT3 and STAT5 was reported to impact chromatin remodeling and gene regulation. Furthermore, the impaired activation of STAT5b seems to be involved in the development of leukemia induced by BCR/ABL (breakpoint cluster region-Abelson oncogene) [30].

Numerous fusion genes were defined as key agents in leukemia development, such as the TEL (Translocation ETS Leukemia)—JAK2 fusion gene that was identified in 1997. Other fusion genes are PCM1-JAK2 in acute myeloid leukemia, in atypical chronic myeloid leukemia and T-cell precursor acute lymphoblastic leukemia (TPALL), ETV6-JAK2 in acute lymphoblastic leukemia (ALL) and in chronic myeloid leukemia (CML) and BCR-JAK2 in atypical chronic myeloid leukemia (aCML) [31].

## 4. JAK-STAT Pathway Inhibitors

### Approved Inhibitors

There are three ways of targeting JAK/STAT signaling either through antibodies targeting cytokines or their membrane receptors, through JAK inhibitors or through STAT inhibitors. Drugs are mostly used to treat autoimmune disorders and hematological cancers, but also solid cancers. Several drugs have been approved, but most of them are still under development. Table 1 summarizes the molecules targeting the JAK-STAT signaling pathway with a focus on their use in oncology.

***JAK-inhibitors or jakinibs***, exert their function by binding to JAK kinase domains. 12 JAK inhibitors have been approved by the FDA, all in the field of autoimmune diseases (Delgocitinib, Ruxolitinib, Fedratinib, Pacritinib, Baricitinib, Pefcitinib, Abrocitinib, Oclatinib, Upadacitinib, Filgotinib, Tofacitinib, Deucravacitinib) [32]. Among these, JAK2 inhibitors are the most widely represented. First-generation (type I) JAK2 inhibitors, such as Ruxolitinib, Fedratinib or Pacritinib, interact with the active conformation of the JH1 kinase domain, blocking the ATP-binding site and thus the phosphorylation of their partner substrates [33]. Nevertheless, these inhibitors do not selectively discriminate between mutated and wild-type forms, and this lack of specificity for tumor cells leads to significant side-effects, particularly hematological toxicities. In addition, JAK2 blockade favors the selection of resistant clones that reactivate JAK/STAT signaling and drive tumor progression.

To counter this, a new-generation of JAK2 (type II) inhibitors has been developed. These inhibitors interact selectively with the inactive conformation of the kinase domain, which differs according to whether the mutated or wild-type form of JAK2 is considered. This provides them with a greater specificity. Finally, selective inhibitors of the JH2 pseudokinase domain are also being developed, which are also effective against mutated and wild-type JAK2.

Moreover, the ubiquitous nature of STATs and their close biological activity, make their inhibition challenging. For example, in response to IL6-mediated signaling, activation of STAT1 can render STAT3 inhibition ineffective. Nevertheless, STAT3 targeting remains the most explored, owing to the fact that it acts as an oncogene when constitutively activated. Different molecules are engaged in the modulation of STAT3 activity by altering various functional domains of this effector. SH2 domain inhibitors interfere with STAT3 phosphorylation and dimerization [34]. DNA-binding domain (DBD) inhibitors, notably DNA-decoy oligonucleotides, competitively prevent STAT3 from binding to promoter sequences of target genes [35]. This is the case, for example, with Napabucasin. N-terminal domain (NTD) inhibitors affect STAT3 dimerization and nuclear translocation, as well as its interaction with the DNA of its target genes [36]. Finally, techniques have been developed to inhibit STAT3 by inducing degradation of its messenger RNA via RNA interference [37].

***Antibody blockade of cytokines and their receptors*** have been extensively explored in the treatment of inflammatory diseases, notably the blockade of IL6 or its receptor. As high levels of IL6 have been reported in the microenvironment of several tumor types including breast carcinomas [38] with a poor prognosis, current studies focus on the use of such molecules as JAK-STAT pathway modulators in cancer treatment. So far, no robust data support their use in routine clinical practice.

**Table 1:** **Overview of JAK-STAT pathway inhibitors approved or under development in oncology: indications, mechanisms of action and safety profile.**

Inhibitors of the JAK/STAT Signaling Pathway in Oncological Trials				
Drug	Mechanism	Approved Indications	Trials in Oncological Treatment	Safety Profile
**RUXOLITINIB** (Jakafi^®^) INCB018424 INC424	Inhibitor of **JAK1** **JAK2** Mutationally activated JAK2 (**JAK2V617F**)	Myelofibrosis Polycythemia vera Acute and chronic graft versus host disease	**Pancreatic cancer**: although promising data from the RECAP phase II trial (2015) indicated that Ruxolitinib may have been approved as a second line treatment of refractory pancreatic cancer with systemic inflammation (CRP > 13mg/dL) progressing after first-line Gemcitabine [39], it failed to show efficacy in two larger phase III trials (JANUS 1 and JANUS 2-2018) [40]. Preclinical studies suggest that combining MEKi with STAT3i via JAK inhibition and immune checkpoint inhibitors is a promising treatment option in pancreatic adenocarcinoma, as it might overcome treatment resistance by mitigating stromal inflammation and reprogramming the tumor microenvironment. A phase I trial is ongoing (NCT05440942) [41]. **Head and Neck cancer (HNSCC)**: Ruxolitinib was found to inhibit tumor growth in HNSCC by inhibiting STAT3 activation, in preclinical models (murine models bearing patient-derived xenografts). STAT3 levels seem to be a promising predictive biomarker for response to Ruxolitinib [42]. A phase II trial evaluating Ruxolitinib efficacy and safety in HNSCC patients that have operable disease, in the neoadjuvant setting, was performed—the data analysis is pending [43]. **Hematological malignancies**: numerous phase I/II trials are ongoing testing Ruxolitinib in MPN. AML, ALL, CLL, T-cell large granular lymphocytic leukemia. The clinical trial (NCT02092324) on chronic lymphoblastic leukemia (CLL) showed an overall response rate (ORR) of 32% among patients. Data from NCT01776723 on chronic monocytic leukemia patients demonstrated favorable survival outcomes and an acceptable safety profile [31]. **Lung cancer**: in a phase II trial, Ruxolitinib showed an acceptable safety profile in association to Cisplatin and Pemetrexed in the first line treatment of advanced NSCLC with systemic inflammation, but no efficacy [44].	Anemia Thrombocytopenia Neutropenia Hypokalemia Infections Peripheral edema
**PACRITINIB** (Vonjo^®^) DB11697	Inhibitor of **JAK2**, mutationally activated JAK2 (**JAK2V617F**), **FLT3**	Myelofibrosis	**Hematological malignancies**: Pacritinib is an approved drug for myelofibrosis (MF), with proven efficacy in symptom control in the phase III PERSIST-1 trial and in NCT01437787. Phase I/II clinical trials are ongoing to test its use in leukemias [31]. **Solid tumors harboring the 1q21.3 copy number amplification**: Chromosome 1q21.3 copy number amplification is associated with IRAK1 upregulation, promoting in vitro tumor cell growth. Additionally, IRAK1 inhibition seems to modulate immune cell populations both in systemic circulation and in the tumor microenvironment. Therefore, Pacritinib that has a known inhibitory effect on IRAK1, is a promising therapeutic agent in this subgroup. The phase Ib/II NCT04520269 trail tested this hypothesis. Although the initial results showed no objective response, dose expansion is ongoing, with potentially favorable results [45]. **Squamous cell lung cancer**: in vitro studies show that Pacritinib effectively inhibits glucose consumption by cancer cells, and does not interfere with normal cell metabolism, therefore potentially blocking cancer cell growth. This effect is obtained by FLT3 inhibition [46]. **Triple negative BC**: in preclinical studies, simultaneously blocking JAK-STAT and SMO-GLI1/tGLI1 by combining Pacritinib to Sonidegib showed a reduction in metastatic potential in an animal model (murine model obtained by the injection of luciferase-expressing MDA-231 cells into the left ventricle) [47].	Diarrhea Prolonged QT interval Peripheral edema Thrombocytopenia
**FEDRATINIB** (Inrebic^®^) TG101348	Inhibitor of **JAK2 JAK2V617F**	Myelofibrosis	**Pancreatic cancer**: a study integrating bioinformatics resources demonstrated that KRAS-driven gene signature plays a crucial role in disease progression and prognosis, and that Fedratinib might be able to reverse the KRAS-driven gene signature, therefore establishing promising therapeutic benefits in this subgroup of patients [48].	Fatal encephalopathies Anemia Gastrointestinal symptoms Increased liver transaminases, creatinine and pancreatic enzymes
**TOFACITINIB** (Xeljanz^®^) CP690550	Inhibitor of **JAK1** **JAK2** **JAK3**	Rheumatoid arthritis Psoriatic arthritis Ankylosing spondylitis Juvenile idiopathic arthritis Ulcerative colitis	**Hematological malignancies:** Tofacitinib showed promising results in treating T-ALL in preclinical trials [32].	Infections Malignancies Anemia, Neutropenia Elevated creatinine and transaminases Hypercholesterolemia Gastrointestinal symptoms Thromboembolism
**BARICITINIB** (Olumiant^®^) LY3009104	inhibitor of **JAK1** **JAK2**	Rheumatoid arthritis SARS-CoV2 infection	**Prostate cancer:** The use of Bacritinib has synergic effects with Docetaxel in castration-resistant prostate cancer in a preclinical trial [49].	Infections Malignancies Hypercholesterolemia
**UPADACITINIB** (Rinvoq^®^) ABT-94	inhibitor of **JAK1**	Rheumatoid Arthritis (AR) atopic dermatitis (AD) Ulcerative Colitis Psoriatic Arthritis	Upadacitinib may offer protection against Cisplatin toxicity to the liver and kidneys, and does not limit its efficacy in the treatment of lung and BC in a preclinical in vitro and in vivo trial (murine model). Nonetheless, its antitumor effect has not been tested [50].	Infections Malignancies Hypercholesterolemia Increased CPK Increased liver enzymes Thromboembolism
**PEFICITINIB** (Smyraf^®^) ASP015K	Pan-JAK inhibitor **JAK1** **JAK2** **JAK3** **TYK2**	Rheumatoid Arthritis (Japan)	**Ovarian cancer**—in vitro studies using ovarian cancer cell lines with knockdown or overexpression of CHAF1A (chromatin assembly factor 1 unit A) that induces cell proliferation and growth, showed an inhibitory effect of Peficitinib [51].	Infections Malignancies Hypercholesterolemia Increased CPK Elevated Creatinine levels
**DEUCRAVACITINIB** Sotyktu™) BMS986165	inhibitor of **TYK2**	Moderate to severe plaque psoriasis	**NF-1 associated malignant peripheral nerve sheath tumors (MPNST)**—Delucravatinib associated with Mirdametinib, a MEK-inhibitor, showed an inhibitory effect on cell proliferation and induced apoptosis in vitro [52].	Upper respiratory infection Folliculitis Mouth ulcers Acne Elevated blood CPK l
**DELGOCITINIB** (Corectim^®^) JTE052	Pan-JAK inh **JAK1** **JAK2** **JAK3** **TYK2**	Atopic Dermatitis (Japan)	-	Upper respiratory tract infections Kaposi’s varicella Contact dermatitis Acne
**ABROCITINIB** (Cibinqo^®^) PF04965842	inhibitor of **JAK1** **JAK2**	Atopic dermatitis (AD)	-	Upper respiratory tract infections Nausea and vomiting Acne, Herpes zoster Increased blood CPK
**FILGOTINIB** (Jyseleca^®^) GLPG0634	inhibitor of **JAK1**	Rheumatoid Arthritis	-	Infections Headache
**OCLACTINIB** Apoquel^®^ PF03394197	inhibitor of **JAK1**	Canine allergic dermatitis	-	Diarrhea Nausea et vomiting Anorexia Fatigue
**STAT inhibitors**				
**NAPABUCASIN** BBI608	inhibitor of **STAT3**	No approved clinical indications. Trails are ongoing	**Pancreatic cancer**: in the phase 3 CanStem111P study (NCT02993731), the addition of Napabucasin to nab-paclitaxel with gemcitabine did not improve efficacy in the treatment of naive metastatic pancreatic adenocarcinoma [53]. **Colorectal cancer**: in the CanStem303C phase III clinical trial, Napabucasin + FOLFIRI versus FOLFIRI is tested for the treatment of previously treated metastatic colorectal cancer. The aim of the study is to objectify the sensitizing effect to chemotherapy by Napabucasin on cancer cells. The results are pending. **Glioblastoma:** Napabucasin induces cell cycle arrest and apoptosis, impairing glioma growth in in vivo xenograft murine models [54].	Fatigue Nausea Diarrhea Anorexia Bone loss
** OPB111077 **	inhibitor of **STAT3**		**Diffuse Large B-Cell Lymphoma (DLBCL**)—in a phase I trial, OPB-111077 in combination with Bendamustine and Rituximab showed promising results in terms of efficacy and safety [55].	Fatigue Nausea Vomiting
** TTI101 **	inhibitor of **STAT3**		**Advanced, treatment-refractory solid tumors**: in a phase I trial (NCT03195699), TTI-101 monotherapy showed promising antitumor activity in relapsed and refractory solid tumors, particularly in the treatment of HCC, while bearing an acceptable safety profile [56] **Advanced hepatocarcinoma**: a phase Ib/II trail (EVERT-Liver Cancer trial, NCT05440708) is ongoing. It will assess the overall response rate and the incidence of adverse effects of TTI-101 use, both in monotherapy and in combination with Pembrolizumab and with Atezolizumab/Bevacizumab [57]. **Metastatic hormone-receptor positive, HER2-negative BC**: The phase Ib/II REVERT-BC trail (NCT05384119), evaluates the association of TTI-101 to AI or Fulvestrant + CDK4/6 inhibitors (Palbociclib). It aims to show the antitumor activity of TTI-101 by reverting resistance to hormone therapy [58].	Diarrhea Hyperglycemia
**DANVATIRSEN** AZD9150	inhibitor of **STAT3**		**Head and Neck cancers (HNSCC)**: in the phase Ib/II SCORE trial, Danvatirsen in association to Durvalumab was superior to Durvalumab alone or Durvalumab + AZD5069 (CX2i) in patients with recurrent or metastatic HNSCC [59]. **Diffuse Large B-Cell Lymphoma**: in a phase I trial, Danvatirsen + Acalabrutinib showed an acceptable safety profile, but no gain in efficacy [60].	Fatigue Thrombocytopenia Increased blood levels of liver enzymes
** BP1102 **	inhibitor of **STAT3**		**T-cell acute lymphoblastic leukemia**: in a preclinical trail, BP-1-102 induced apoptosis and cell cycle arrest in T-ALL cell lines [61].	Toxicity profile not known 4
**Antibody blockade of cytokines and their receptors**				
** TOCILIZUMAB ** (Actemra^®^) L04AC07	humanized monoclonal antibody targeting **IL6-R**	Rheumatoid arthritis Giant cell arthritis Cytokine release syndrome	**Epithelial ovarian cancer:** in the NCT01637532 phase I trial, Tocilizumab in association with interferon-α2b and carboplatin/doxorubicin showed a good safety profile. Its antitumor activity remains to be explored in a following phase II trial [62]. **Renal cell carcinoma**: in preclinical in vitro and in vivo (xenograft) studies, Tocilizumab significantly suppressed cell proliferation, due to its capacity to suppress SOCS3 expression, a negative regulator of the JAK-STAT pathway [63].	Upper respiratory tract infections Headache Increased blood pressure Injection site reactions
** SILTUXIMAB ** (Sylvant^®^) CNTO328	chimeric monoclonal antibody targeting **IL-6**	HIV-negative and HHV-8-negative multicentric Castleman’s disease	**Hematological malignancies**: Siltuximab was explored in the treatment of refractory Multiple Myeloma, in multiple phase II trials, but it failed to show an antitumor activity. Siltuximab failed to reduce blood transfusions in low and intermediate risk MSD [64] **Solid tumors**: in a phase I/II trail, Situximab monotherapy seems to be safe, but with no clinical activity in advanced solid tumors [64].	Anaphylactic reaction at infusion Skin rash Pruritus Upper respiratory tract infection Weight gain Increased blood level of uric acid

## 5. JAK/STAT in Solid Tumors

The JAK/STAT pathway is also involved in carcinogenesis and therapeutic resistance in solid tumors, mainly through the involvement of STAT3. Tumor development due to STAT3 aberrant activation results from loss of competent immune signaling and by the induction of a pro-inflammatory response in the tumor microenvironment. Growth factors and cytokines (IL-6, IL-7, IL-10, GM-CSF, VEGF, TGF-β) are produced by the surrounding stroma and also by tumor cells, and induce, by coupling to their cell membrane receptors, JAK-STAT3 activation. STAT3 subsequently transduces the signal, resulting in IL-6 and IL-10 production, therefore amplifying this chronic pro-inflammatory loop and inducing tumor growth [65].

Furthermore, one of the major drawbacks of the current drugs used in oncology is the development of resistance by cancer cells. This resistance is conferred by cell plasticity, driving a shift in cell phenotype towards a refractory state, that can sometimes be acquired by reverting back to a stem cell-like state and cell redifferentiation into alternative lineages, or through a direct transdifferentiation into a different lineage. The mechanism underlying this process is not fully elucidated, though it seems to revolve around STAT3 signaling. In prostate cancer, JAK-STAT plays a primary role in cell lineage plasticity, allowing lineage conversion from a luminal to basal phenotype, and even cell differentiation into squamous cell carcinoma or neuroendocrine carcinoma, therefore resulting in drug resistance.

In addition, constitutive aberrant STAT3 activation promotes cell migration and invasion. In vitro studies showed that genetic (KO cells for JAK1/JAK2 and STAT1/STAT3) and pharmacological inhibition of the JAK-STAT pathway might suppress resistance to anti-androgen receptor therapies by reducing lineage plasticity [66]. In head and neck squamous cell carcinomas, related or not to a prior HPV infection, STAT3 hyperactivation was associated with worse prognosis, as it drives cell growth and resistance to both cytotoxic drugs and anti-EGFR antibodies. Furthermore, constitutive STAT3 activation drives immunosuppression, thus allowing cancer evasion from cytotoxic T-lymphocytes [67].

In hepatocarcinoma (HCC), STAT3 is a pivotal oncogenic driver by transcriptionally activating genes involved in hepatocarcinogenesis, promoting cell cycle progression, angiogenesis, immunosuppression and inhibiting apoptosis. It is constitutionally activated in up to 60% of HCC patients. A major consequence of the activation of this signaling pathway is the release of cytokines by diverse inflammatory cells that surround the tumor. Furthermore, STAT3 activation seems to play a crucial role in the development of resistance to Sorafenib, a multi-tyrosine kinase inhibitor used in clinical practice to treat advanced HCC, by the regulation of an anti-apoptotic protein, Mcl-1 [66].

### 5.1. Focus on JAK/STAT Involvement in Breast Cancers

BC is a molecularly diverse disease that can be categorized into three main groups based on molecular and histological evidence: hormone receptor-positive (HR+), human epidermal growth factor receptor 2-positive (HER2+), and triple-negative BC (TNBC). The subdivision of BC into HR+, HER2+ and TNBCrs is fundamental in current practice, as it gives rise to different treatment proposals. In recent years, the prognosis of HR+ BC has been significantly improved by the emergence of CDK4/6 inhibitors in adjuvant and metastatic settings, in combination with hormone therapy [68,69,70,71,72,73,74,75].

The development of antibody drug conjugates (ADCs) such as Trastuzumab Deruxtecan or Sacituzumab Govitecan has also led to a significant improvement in progression-free survival (PFS) and even overall survival (OS) in the metastatic phase [76,77]. These molecules are particularly attractive alternatives to standard chemotherapy. The prognosis of HER2+ cancers has also been revolutionized by the development of increasingly effective treatments targeting HER2, such as ADCs (e.g., Trastuzumab Deruxtecan and Trastuzumab Emtasine) [78,79,80] or tyrosine kinase inhibitors (such as Tucatinib in combination with Trastuzumab and Capecitabine) [81].

Finally, TNBC management have also benefited, to a lesser extent, from interesting therapeutic innovations with the advent of Pembrolizumab in combination with chemotherapy in localized situations (irrespective of PDL1 status) [82] or as a first-line metastatic treatment (for tumors with a PDL1 combined positive score > 10%) [83]. For subsequent lines of treatment, ADCs like Sacituzumab Govitecan [84] and Trastuzumab Deruxtecan [85] have led to significant survival benefits. In the subset of patients with a germline BRCA1/2 mutation, PARP inhibitors were shown to affect both localized (overall survival benefit) [86] and metastatic (progression-free survival benefit) [87,88] stages. Nevertheless, despite this overall improvement in the prognosis of BC, irrespective of the subtype, the outcome once patients reach the metastatic stage is almost always unfavorable, due to the emergence of clones resistant to standard therapies.

As previously mentioned, the JAK/STAT pathway is implicated in therapeutic resistance, as well as in oncogenesis and metastatic dissemination of many cancer types, including BC [89]. The literature shows that JAK mutations are rare in BC. Indeed, a PCR-single strand conformation polymorphism (SSCP) assay revealed 3 mutations in 90 BC cases: 1 mutation in JAK1 c.1939C>T (p.H647Y), 1 mutation in JAK3 c.2143G>A (p.V715I) and 1 mutation in JAK3 c.420+28G>A (unknown) [90]. This was confirmed by the analysis of 816 cases of TCGA-derived BCs, identifying JAK alterations in a limited number of cases. For JAK1, 2 truncating mutations, 2 splice mutations, 5 VUS, 10 amplifications and 3 deletions were found. For JAK2, 7 VUS, 19 amplifications and 6 deletions were found. For JAK3, there were 7 VUS and 14 amplifications. For TYK2, there were 6 SUVs and 18 amplifications [91].

Nevertheless, despite this low rate of mutations and copy number variation in JAKs, aberrant activation of the JAK/STAT pathway is particularly prominent in BC, notably in TNBC and HR+ BCs. Indeed, a conditional KO in mice showed that JAK2 is involved in the proliferation and differentiation of alveolar cells of the mammary gland and their maintenance during lactation [92]. It appears that JAK2 associated with STAT5, STAT3 and STAT1 signaling is preferentially involved in the development, progression and metastasis in these 2 subtypes, as well as in their resistance to treatment.

The role of STAT5 in breast carcinogenesis is controversial. Indeed, Cotarla et al. found a nuclear expression of p-STAT5 in 76% of cases studied in their series [93]. However, in several studies, such expression is correlated with a good prognosis [94,95]. The pro-differentiation and anti-invasion properties of STAT5 reported by Sultan et al. could explain this good prognosis [96]. Nevertheless, other studies have clearly established the tumorigenic role of STAT5. Some teams have also shown that hyperactivation of STAT5 in caveolin1 KO mice can lead to the formation of hyperplasia and even well-differentiated mammary carcinomas via increased expression of ERα and Cyclin D1 [97,98].

In addition to Cyclin D1, other genes involved in cell cycle progression, such as myc, can be upregulated by STAT5, thus participating in tumorigenesis [99]. Iavnilovitch et al. showed in mouse models an association between STAT5 overexpression and the formation of well-differentiated papillary or micropapillary breast adenocarcinomas [100]. Caffarel et al. demonstrated the impact of JAK2/STAT5 activation in mammary gland tumorigenesis [101], as V617F JAK2 overexpression activated STAT5, thus triggering cell proliferation and resistance to apoptosis in in vitro mammary epithelial cell models, as well as tumorigenesis in mouse models. Finally, Rädler et al. showed that in mammary epithelial cells, the JAK2/STAT5 pathway controls mammary epithelial cell survival and death through the direct interaction with the p58α regulatory subunit of PI3K and upregulation of the expression of p85α (Pik3r1), p110α (Pik3ca), and AKT1 [102]. Interestingly, STAT5 activation can drive both proliferative and anti-apoptotic signals, which is critical for hormone receptor-positive BC cells resistant to endocrine therapy [103,104].

STAT3 activation has been described in vitro and in vivo in cell models and primary BC tissues [105,106]. It is associated with an unfavorable prognosis [2,107,108], notably by driving resistance to anticancer therapies. For example, an ancillary study of 45 patients treated for localized BC reported an inverse correlation between STAT3 activity and the rate of complete histological response to neoadjuvant chemotherapy with Docetaxel and Doxorubicin [105]. On the other hand, a recent publication showed that ZIP (for Zinc finger and G-patch domain-containing protein), a transcriptional repressor is able to decrease JAK2 expression by binding to its promoter. In BC cells, the absence of ZIP induces an increase in JAK2 expression and the downstream P-STAT3 activation was associated with resistance to Tamoxifen [109].

In BC, STAT3 activation can be both constitutive and transient, contributing to tumorigenesis in different ways. Constitutive activation of STAT3 (pSTAT3), a hallmark of many cancers, is driven by persistent exposure to cytokines such as IL-6, IL-10, and oncostatin M, leading to continuous phosphorylation and nuclear translocation of STAT3. Once activated, STAT3 induces the expression of genes such as *Bcl-xL*, *Mcl-1*, and *Cyclin D1*, which promote cell survival, proliferation, and resistance to apoptosis [99]. This chronic activation helps maintain cancer stem cell populations and facilitates epithelial–mesenchymal transition (EMT), key drivers of metastasis and recurrence [103,110,111]. Constitutive STAT3 activation also contributes to immune evasion. By upregulating immune checkpoint proteins like PD-L1, STAT3 allows cancer cells to escape immune surveillance, which is a major factor in therapeutic resistance, particularly in TNBC [10].

In addition to its constitutive role, STAT3 can also be transiently activated in response to acute stimuli, including cytokine signaling and cellular stress from therapies like chemotherapy or radiation. This transient activation enables BC cells to mount a short-term survival response by upregulating anti-apoptotic genes such as *Bcl-xL* and *Mcl-1* [112]. Although this activation is temporary, it allows cancer cells to evade treatment-induced cell death, contributing to the development of resistance [99]. A growing body of evidence shows that the JAK/STAT pathway interacts with other critical signaling cascades in BC, such as the PI3K/AKT/mTOR and NF-κB pathways [113]. In TNBC, this cross-talk contributes to the inflammatory microenvironment and enhances the proliferation and migration of cancer cells. Activation of NF-κB can enhance JAK/STAT signaling by promoting cytokine secretion (e.g., IL-6), which further drives STAT3 activation. This cross-talk between pathways is prompting studies into their combined targeting in future therapies [113].

**Figure 2:**
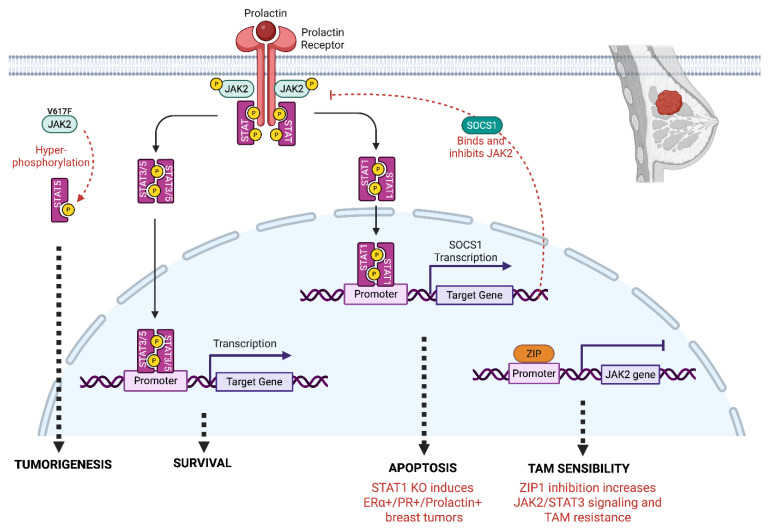
**Role of JAK2 signaling in breast cancer.** JAK2 activates STAT1 and STAT3/5 through phosphorylation. Among the target genes regulated, the JAK2/STAT1 pathway induces the expression of SOCS1 that negatively regulates JAK2 signaling. In mice STAT1 KO, the negative control of JAK2 is absent, inducing survival and luminal breast tumors. ZIP (Zinc finger and G-patch domain containing protein) is a transcriptional repressor that binds to the JAK2 promoter and negatively regulates JAK2 expression. When ZIP1 is absent, the expression of JAK2 increases, inducing JAK2/STAT3 signaling and resistance to Tamoxifen (TAM). The overexpression of the constitutively active mutant JAK2 (JAK2^V617F^) triggers a hyperactivation of STAT5, inducing breast tumorigenesis.

The role of STAT1 is more controversial, in some studies it plays an oncogenic role, while others consider it as a tumor suppressor. In the study by Chan et al. 45% (37/83) of ER+ tumors and 22% (17/78) of ER− tumors showed low IHC expression of STAT1 compared to adjacent peri-tumoral tissues, suggesting that loss of STAT1 expression is involved in tumor progression [114]. However, the same authors demonstrated that the knock down of STAT1 induces luminal breast tumors in mice [114], which is correlated with the frequent loss of STAT1 in ERα-positive BC. Mechanistically, this can be explained by the fact that while prolactin triggers STAT3 and STAT5A/B activation via JAK2 to promote cell proliferation and survival, JAK2 also activates STAT1 which triggers SOCS1 transcription. In the absence of STAT1, the negative control on STAT3 signaling is absent, favoring the development of breast tumors [115] (Figure 2). These data, establishing STAT1 as a tumor suppressor, are substantiated by those of Widschwendter et al. [116] who correlated STAT1 expression and activity with a more favorable prognosis, notably in TNBC and HER2+ subtypes.

Interestingly, other studies have highlighted the pro-oncogenic role of STAT1. For example, Weichselbaum et al. associated STAT1 expression with resistance to cytotoxic-mediated DNA damage [117]. The same team demonstrated the involvement of MUC1 in these phenomena and highlighted in two independent BC databases the unfavorable prognostic role of STAT1 and MUC1 co-expression on relapse-free survival and overall survival [118]. Some authors have suggested that the function of STAT1 as an oncogene or tumor suppressor may be dependent on menopausal status. Indeed, p-STAT1 expression has been associated with an unfavorable prognosis in terms of overall survival (OS) in premenopausal patients and with a favorable prognosis in terms of disease-free survival in the postmenopausal women specifically those harboring HR+ tumors [119].

### 5.2. Inhibitors Under Development to Target Breast Cancer

**Ruxolitinib** is the most extensively studied JAK1/2 inhibitor in BC. In TNBC models, it reduces IL-6-mediated STAT3 activation, which in turn downregulates genes involved in survival and metastasis, such as *Bcl-xL*, *VEGF*, and *MMP9* [120]. In a phase II study conducted in a second-line hormone therapy setting in patients with hormone receptor-positive, HER2-negative breast cancer, the use of Ruxolitinib in combination with exemestane resulted in a clinical benefit rate of 24% in the overall population (95% CI 9.4–45.1), with 6/25 patients experiencing stable disease for more than 6 months. However, the median PFS was only 2.8 months in this study (95% CI 2.6–3.9), possibly due to modest on-target inhibition of phosphorylated STAT3 by ruxolitinib at tolerable dose [121].

Similarly, its use in the treatment of triple-negative breast cancer was not associated with any significant clinical benefit. In a phase II study evaluating Ruxolitinib in patients with refractory metastatic triple-negative breast cancer, none of the 21 patients who received at least one dose of treatment showed an objective response, leading to premature discontinuation of the study due to futility. A possible on-target effect in some patients was suggested by a significant decrease after treatment in the percentage of pSTAT3-positive cells on immunohistochemistry and in the expression of JAK-STAT pathway target genes on whole transcriptome RNA sequencing. Nevertheless, the intratumoural heterogeneity of pSTAT3 and JAK2 amplification in pre- and post-treatment biopsies could explain the lack of efficacy of Ruxolitinib as monotherapy [108]. The response was better when Ruxolitinib was used in combination with other therapies. In a phase 2 trial, Ruxolitinib was combined with Capecitabine (precursor of 5 fluorouracil) in HER2^−^ advanced BC. The objective response rate (ORR) was 28.9% in the Ruxolitinib, compared to 13.7% in the placebo group, showing a significant increase in tumor shrinkage for patients receiving the combination therapy, particularly in patients with high baseline inflammation. However, neither OS nor PFS was improved compared with placebo plus Capecitabine [122].

**Momelotinib**, another JAK1/2 inhibitor, has shown preclinical efficacy in TNBC models. By inhibiting JAK1/2 and suppressing IL-6/STAT3 signaling, Momelotinib reduced tumor growth and metastasis. In TNBC xenograft models, treatment with Momelotinib resulted in a 60% reduction in tumor volume compared to controls, underscoring its potential to reduce cancer stem cell populations and enhance sensitivity to chemotherapy [2].

**Tocilizumab**, an IL-6 receptor antagonist, represents an indirect strategy to inhibit JAK/STAT signaling. By blocking the IL-6 receptor, Tocilizumab prevents the downstream activation of STAT3, which is particularly relevant in BCs driven by IL-6-mediated inflammation. Preclinical studies in TNBC models showed that Tocilizumab reduced STAT3 activation by 50%, resulting in decreased tumor cell proliferation and metastasis [99,103].

### 5.3. Other Inhibitors in Preclinical Development

Several other JAK inhibitors are under preclinical investigation for BC treatment. **Pacritinib** and **Fedratinib**, both JAK2-selective inhibitors that display efficacy in myeloproliferative disorders, are being investigated for their relevance in solid tumors like BC and have shown promising results in reducing STAT3 activity and tumor progression in TNBC models. Pacritinib, for instance, reduced metastasis by 40% in preclinical TNBC mouse models, though its clinical development has been limited by concerns over toxicity and off-target effects [104]. Despite promising results from JAK inhibitors like Ruxolitinib and Momelotinib, their clinical use in BC has been tempered by limited responses and resistance mechanisms. Combination strategies, such as pairing JAK inhibitors with PI3K/AKT inhibitors or immune checkpoint inhibitors (e.g., anti-PD-L1 therapies), could enhance their efficacy by overcoming compensatory pathways [10]. Furthermore, identifying biomarkers such as elevated IL-6 levels or specific STAT3 gene signatures may help stratify patients who are more likely to benefit from these therapies [123].

## 6. JAK/STAT Signaling and Resistance to Treatment

### 6.1. Proteomic Studies of the JAK/STAT Pathway in Cancer

**Figure 3:**
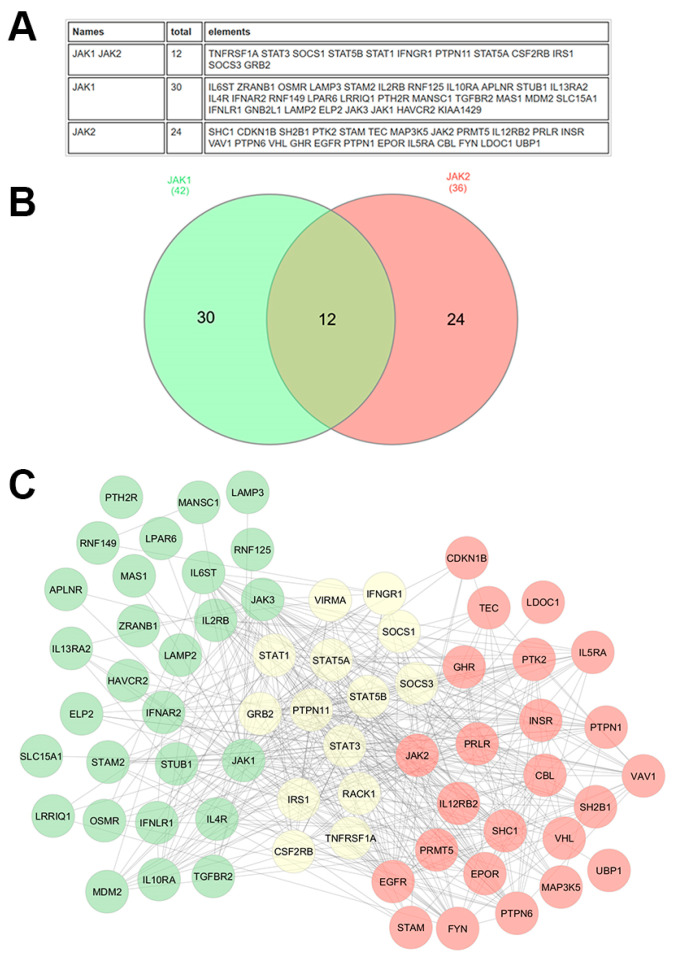
**JAK1 and JAK2 interactome.** The human JAK1 and JAK2 interactome was derived from BioGRID, including all physical interactions supported by at least two independent references, encompassing both low- and high-throughput experimental evidence. The data are presented in two formats: (**A**) as a comprehensive table listing the interacting proteins, and (**B**) as a Venn diagram presenting the overlap of interactors between JAK1 and JAK2. (**C**) Interaction networks were further analyzed using experimental data from the STRING database and visualized in Cytoscape (3.10.3). In the Cytoscape network, green nodes represent proteins that specifically interact with JAK1, red nodes represent proteins that interact specifically with JAK2, and yellow nodes represent proteins that interact with both JAK1 and JAK2. Edges indicate experimentally validated physical interactions. This analysis highlights both shared and unique components of the JAK1 and JAK2 interactomes, providing insights into their overlapping and distinct functional roles.

Proteomic approaches offer a deeper understanding of protein-level alterations, and protein interactions that drive oncogenesis. Proteomics, which involves the large-scale study of proteins, has been instrumental in revealing key aspects of the JAK/STAT pathway in cancer. The use of mass spectrometry (MS)-based proteomics, in particular, has allowed the identification and quantification of proteins involved in this pathway. Figure 3 is an example of the interactors common and specific to JAK1 and JAK2, highlighting that both proteins display specificities.

Proteomics has also played a key role in understanding resistance to JAK inhibitors, such as Ruxolitinib, which is commonly used to treat MPNs. By comparing the proteomes of sensitive and resistant cells, researchers have uncovered compensatory activation of alternative signaling pathways [124]. Specifically, the PI3K/AKT and MAPK pathways are frequently upregulated in resistant cells, helping to sustain cell survival and proliferation despite JAK inhibition. These compensatory pathways are believed to contribute directly to the development of resistance, suggesting that targeting these additional pathways alongside JAK inhibitors could improve treatment efficacy for MPNs (Figure 3).

In solid tumors, aberrant activation of the JAK/STAT pathway, particularly STAT3 and STAT5, has been extensively studied using proteomics approaches [125]. In BC, for instance, STAT3 promotes EMT [126], a process that enhances cancer cell invasiveness and metastasis. Proteomics studies have identified STAT3-interacting proteins that drive EMT, shedding light on potential therapeutic targets aimed at inhibiting cancer progression [127].

The link between STAT3 interactions, EMT and PRMT1 lies in their roles in cancer progression and immune modulation. STAT3, activated by pathways such as EGFR and IL-6/JAK interacts with multiple proteins, including PRMT1, which facilitates TGFβ-induced EMT through arginine methylation of SMAD7. This EMT process, crucial for cancer metastasis, is consistent with the involvement of STAT3 in immune suppression and tumor microenvironment regulation. Furthermore, proteomics profiling of BC tissues identified STAT3-regulated proteins involved in immune evasion, which may offer new avenues for immunotherapy development [128]. Lung cancer studies have also benefited from proteomics analyses of the JAK/STAT pathway. For example, phosphoproteomics profiling of lung adenocarcinoma cells revealed hyperactivation of STAT3 in response to IL-6 [129], a cytokine abundant in the tumor microenvironment. This finding underscores the role of tumor-derived cytokines in sustaining STAT3 signaling and promoting cancer cell survival. In prostate cancer, proteomics has been used to study the interaction between STAT5 and androgen receptor signaling, revealing a complex network of interactions that drive tumor growth in hormone-resistant cases [130].

### 6.2. Other JAK Substrates

**Figure 4:**
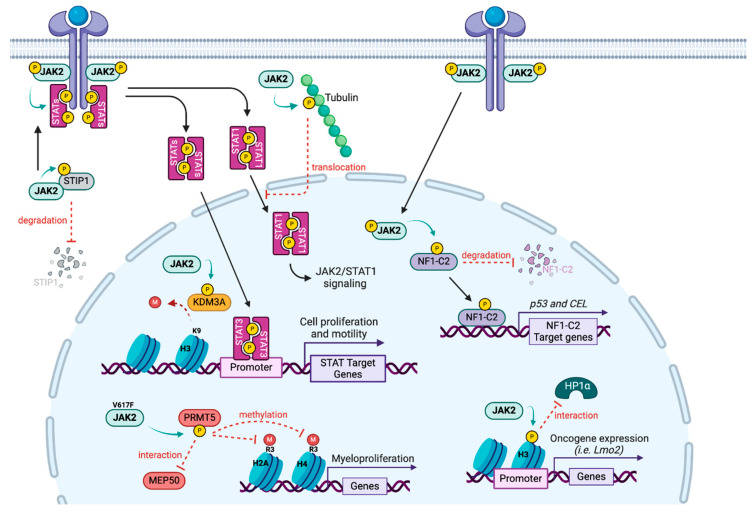
**JAK2 substrates.** Alongside its main target STATs, JAK2 phosphorylates other proteins in the cytoplasm (STIP1, Tubulin) but also in the nucleus (NF1-C2, KDM3A, H3 and PRMT5), leading to a direct transcriptional regulation.

To better understand resistance to JAKi, it is important to note that although STAT proteins are the major substrates for JAK, several substrates have been described, implicating JAK2 protein in other pathways, notably in the nucleus where it can directly regulate transcription (Figure 4). Although JAK2 has previously been observed in the nucleus of several cell types, in 2009, Dawson and colleagues identified the first nuclear substrate by showing that JAK2 phosphorylates the Y41 residue of histone H3, releasing HP1α from chromatin and resulting in the transcription of the LMO2 oncogene [131]. In the mouse mammary gland in the presence of prolactin, JAK2 translocates to the nucleus where it phosphorylates the transcription factor NF1-C2, protecting it from proteasomal degradation. Thus NF1-C2 acts as a coactivator favoring the transcription of p53 and the milk protein carboxyl ester lipase [132]. In Hela cells, IL6 triggers the phosphorylation of KDM3A on the Y1101 residue by JAK2, which increases its demethylase activity, leading to a decrease in the repressive mark H3K9me2 [133]. KDM3A targeting could thus be a potent therapeutic strategy to control the oncogenic effect of the JAK2/STAT3 signaling.

JAK2 also phosphorylates the arginine methyltransferase PRMT5, first identified as janus kinase-binding protein 1 [134]. Although wild type JAK2 interacts with PRMT5, the V617F mutated JAK2 has a higher affinity for PRMT5. The enzymatic activity of PRMT5 is diminished due to a decrease in interaction with its main coactivator MEP50, contributing to the myeloproliferative phenotype [135]. However, the authors only assessed histone methylation, and PRMT5 has since been shown to methylate numerous non histone substrates, suggesting that the impact may be broader than transcriptional regulation.

Other JAK2 substrates have been identified outside of the nucleus. For instance, JAK2 was reported to phosphorylate tyrosine residues and microtubule polymers of tubulin. Upon growth hormone treatment, phosphorylated tubulin participates in the nuclear translocation of STAT1 [136]. In addition, in ovarian cancer cells, JAK2 can phosphorylate STIP1 (stress-induced phosphoprotein 1), a heat shock protein adaptor on Y134 and Y152 residues, maintaining JAK2/STAT3 signaling and interfering with the JAK2/STIP1 interaction, thus leading to cell death [137].

## 7. Conclusions

Currently, JAK/STAT inhibitors are mostly used to treat inflammatory diseases and hematological malignancies. Although most trials are still in a preclinical or early phase, JAK/STAT pathway inhibition is a promising strategy for novel drug development for the treatment of both hematologic and solid malignancies. However, the intrinsic complexity of the pathway needs further clarification to fill knowledge gaps and to improve the manipulation of this cascade of agents. Ongoing efforts to better understand JAK/STAT signaling pathways and their regulation should help identify predictive biomarkers and better target subpopulations of patients prone to respond to therapies targeting key actors of this signaling pathway.

In this context, proteomic studies have significantly advanced our understanding of the JAK/STAT pathway in cancer, providing insights into signaling mechanisms that drive oncogenesis. By employing advanced proteomic techniques such as mass spectrometry and phosphoproteomics, researchers have been able to identify key proteins, PTMs, and interaction networks involved in JAK/STAT dysregulation. These discoveries have important implications for cancer diagnosis, prognosis, and treatment, particularly in identifying new therapeutic targets and understanding resistance to existing therapies. As proteomic technologies continue to evolve, future studies are expected to provide even more detailed insights into the complexities of the JAK/STAT pathway, ultimately improving cancer treatment strategies. In the context of BC, it appears that the JAK2/STAT3 axis is preferentially involved, and knowing more about the regulation of this interaction and other substrates than STAT proteins may help to better target this signaling pathway.

## Data Availability

No new data were created or analyzed in this study.
